# Deep brain stimulation-associated brain tissue imprints: a new in vivo approach to biological research in human Parkinson’s disease

**DOI:** 10.1186/s13024-016-0077-4

**Published:** 2016-01-28

**Authors:** Affif Zaccaria, Ali Bouamrani, Stephan Chabardès, Michèle El Atifi, Eric Seigneuret, Johannes A. Lobrinus, Michel Dubois-Dauphin, François Berger, Pierre R. Burkhard

**Affiliations:** NeuroProteomics Group, University Medical Center, Faculty of Medicine, Geneva, Switzerland; CEA-LETI, Clinatec, Edmond J. Safra Biomedical Research Center, Grenoble, France; Clinique de neurochirurgie, CHU de Grenoble, Grenoble, France; Department of Pathology, Geneva University Hospitals, Geneva, Switzerland; Department of Pathology and Immunology, University Medical Center, Faculty of Medicine, Geneva, Switzerland; Inserm UA01, Clinatec, Edmond J. Safra Biomedical Research Center, CEA, Grenoble CHU, Univ. Grenoble Alpes, Grenoble, France; Inserm, U836, Grenoble Institut des Neurosciences, Univ. Grenoble Alpes, Grenoble, France; Department of Neurology, Geneva University Hospitals, Geneva, Switzerland; Centre Medical Universitaire (CMU), Rue Michel Servet 1, CH-1211 Genève 4, Switzerland

**Keywords:** Parkinson’s disease, Deep Brain Stimulation, Brain Tissue Imprint, Histological analysis, Poly-omic approaches

## Abstract

**Background:**

Deep brain stimulation (DBS) of the subthalamic nucleus (STN) or the internal segment of the globus pallidus (GPi) has been established as a highly effective symptomatic therapy for Parkinson’s disease (PD). An intriguing biological aspect related to the DBS procedure is that a temporary contact establishes between surgical instruments and the surrounding brain tissue. In this exploratory study, we took advantage of this unique context to harvest brain material adhering to the stylet routinely used during surgery, and to examine the biological value of these samples, here referred to as “brain tissue imprints” (BTIs).

**Results:**

Nineteen BTIs from 12 STN- or GPi-electrode implanted patients were obtained in vivo during DBS surgery, without any modification of the surgical procedure. Immunofluorescence analyses confirmed that our approach allowed the harvesting of many neural cells including neurons harboring distinct neurotransmitter markers. Shotgun proteomic and transcriptomic analyses provided for the first time molecular information from DBS-associated brain samples, and confirmed the compatibility of this new type of sample with poly-omic approaches. The method appears to be safe and results consistent.

**Conclusions:**

We here propose BTIs as original and highly valuable brain samples, and DBS-related brain imprinting as a new conceptual approach to biological research in living patients with PD.

**Electronic supplementary material:**

The online version of this article (doi:10.1186/s13024-016-0077-4) contains supplementary material, which is available to authorized users.

## Background

Nearly four decades of intensive research have proved yet unsuccessful at deciphering the aetiology and mechanisms underlying nigral decay in Parkinson’s disease (PD) [1–3], in spite of myriads of hypotheses that have been proposed thus far [4]. Reasons for this ongoing uncertainty are many, including the extensive use of animal models only partly relevant to human PD [5] and the conducting of hypothesis-driven protocols [6, 7] that may be too narrow to disentangle the numerous mechanisms likely at work in PD degeneration. Because hypothesis-driven protocols based on animal models of PD have failed somehow, more global and hypothesis-free strategies have been undertaken in humans, possibly the only source of biological and molecular information truly relevant to PD [8]. Examples include genome-wide association studies [9, 10] or omic approaches [11–13]. In PD, while various peripheral tissues or biological fluids are easily amenable to collection in living patients, relevant neural tissue can be obtained only at autopsy from deceased patients. Indeed, during the last ten years or so, a number of proteomic and transcriptomic studies have been conducted on post-mortem brain tissue samples from various PD-relevant brain structures [11, 14]. However, post-mortem samples are associated with a number of limiting and confounding factors [15–17]. First, difficulties to obtain autopsy tissue [18] tend to keep the number of available samples per study quite low, thus minimizing statistical power to draw firm conclusions. Second, the quality of autopsy tissue is intimately related to the duration and specificities of the pre-mortem agonal state and post-mortem interval (PMI) [19, 20]. Finally, with few exceptions, post-mortem samples are obtained from patients at an advanced stage of disease, making it difficult to identify early molecular events at the basis of the complex basal ganglia (BG) electrophysiology and PD pathogenesis.

To overcome these limitations, and considering all issues discussed above, it may seem that the ultimate sample for biological research in human PD is still to be found. Ideally, it has to be safely and routinely obtained from a large number of living individuals with well-documented sporadic PD. It should consist of biological material directly involved in PD pathogenesis, in this case brain material. Methods to collect samples should be standardized and sampling-to-freezer time should be kept as short as possible.

While this may seem unrealistic at first glance, we propose a novel strategy to obtain PD-relevant samples that may approximate these requirements, based on the concept of in vivo brain tissue imprinting during electrode implantation for deep brain stimulation (DBS) [21]. In fact, we hypothesized that, during this procedure, brain material of interest may adhere to surgical instruments, which transiently interact with implanted nuclei and can be retrieved and analysed at the cellular and molecular level, for example through omic strategies. In this study, we demonstrate for the first time the feasibility, safety, and scientific interest of collecting and studying such DBS-associated samples, here referred to as brain tissue imprints (BTIs), which may provide unique biological signatures of deep brain structures, and, in our opinion, may represent a new paradigm in PD research using highly relevant brain material from living patients.

## Results

### Brain tissue imprinting of BG nuclei

**Fig. 1:**
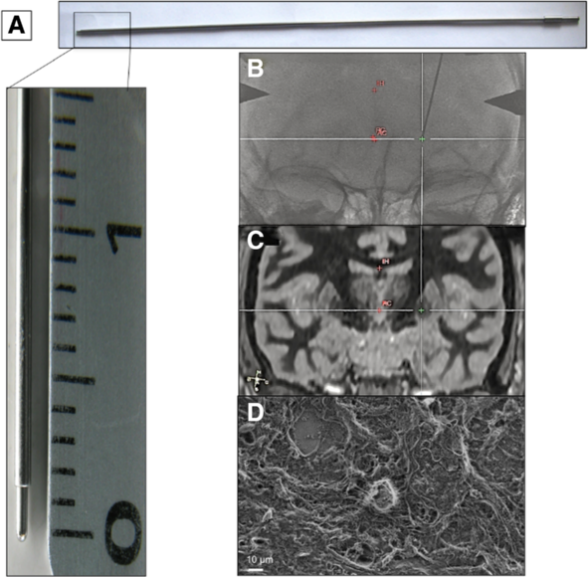
The BTI procedure. **a** The set combines a guide tube and a rigid blunt stylet. The stylet slides through the guide tube and its tip protrudes by 1.5 mm out of the guiding tube. **b-c** The stylet tip is lowered to the targeted nucleus (here the GPi) and interacts with the brain parenchyma according to (**b**) the perioperative radiographic control and (**c**) the pre-operative MRI-defined trajectory. **d** Scanning electron microscopy of the stylet tip-associated tissue revealed the presence of cell bodies and fibers

In this study, 12 patients have been offered bilateral electrode implantation [22] in STN or GPi to alleviate their motor symptoms (Table 1). Once the optimal target site for the chronic electrode to be implanted has been defined, neurosurgeon A or B lowered a blunt stylet inside a guiding tube (Fig. 1a), to the neuroimaging-defined target site (Fig. 1b). First, the radiographic projection on the patient’s preoperative brain MRI confirmed that the stylet tip was within the surgical target, i.e. the STN or GPi (Fig. 1c). The surgeon kept the stylet tip exposed one minute into the patient brain before withdrawing it through the guide tube, and finally removed the set from the brain parenchyma. From the 12 bilaterally implanted patients, a total of 19 stylets was collected and tips that harvested tissue fragments as observed with scanning electron microscopy (Fig. 1d) were immersed in appropriate buffer for further analyses (Table 1). No immediate or late complication from the procedure could be identified. These data demonstrate that the transient contact of the stylet tip with DBS targeted nuclei allows brain material harvesting with no significant modification of the routine DBS procedure.

**Table 1:** Patient information and clinicopathological data of human BTIs

Patients	Gender	Age, yr	Disease duration, yr	Implanted nuclei	Neurosurgeon	BTI number Left (L) or Right (R)	Experiment
P1	F	41	6	STN	A	L1	SEM
						R1	CB-IHC
P2	F	60	10	STN	B	L2	CB-IHC
						R2	CB-IHC
P3	M	52	8	STN	A	L3	P
						R3	T
P4	M	53	7	STN	B	L4	T
						R4	T
P5	M	46	6	STN	A	L5	P
P6	M	54	11	STN	B	R6	P
P7	M	61	9	STN	B	L7	P
						R7	P
P8	M	53	9	GPi	B	L8	P
						R8	P
P9	F	45	10	GPi	A	L9	P
P10	M	69	14	GPi	A	L10	P
P11	F	59	12	GPi	A	L11	P
P12	M	52	11	GPi	B	R12	P

Eight men (M) and 4 women (F) had been offered DBS in STN or GPi by neurosurgeon A or B. BTIs were obtained from left (L) or right (R) hemisphere and used for scanning electron microscopy (SEM), cellblock immunohistochemical (CB-IHC), proteomic (P) or transcriptomic (T) analyses

### Confirming the capture of neural cells by immunohistochemical analyses

**Fig. 2:**
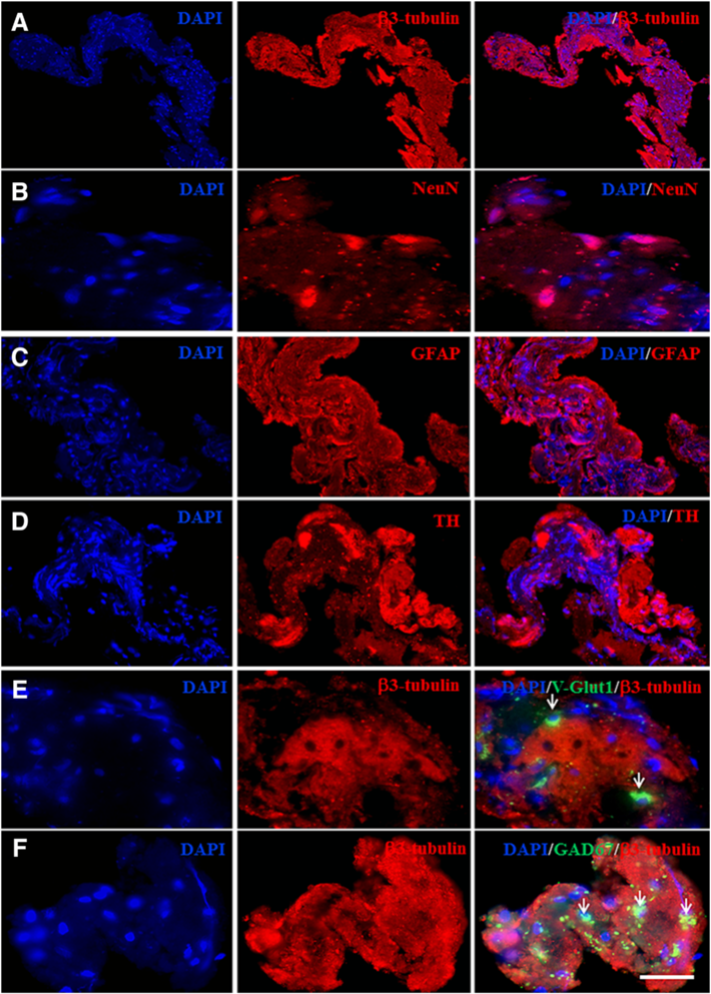
BTI-associated immunohistochemical analyses. Neuronal markers β3-tubulin (**a**) and NeuN (**b**) as well as astroglial marker GFAP (**c**) were identified in BTIs. The presence of tyrosin hydroxylase (TH) was detected in regions poor in cell nuclei, suggesting the presence of TH-immunoreactive axons (**d**). Glutamatergic (**e**) and GABA-ergic (**f**) β3-tubulin immunoreactive neurons were also detected (arrows). Scale bar: A = 160 μm, B = 20 μm, C = 90 μm, D = 85 μm, E and F = 30 μm

To characterize these tissue fragments and confirm the capture of neural cells, three different STN-associated stylet tips were collected in fixative solution for immunohistochemical analyses. First, a hematoxylin-eosin staining revealed the presence of numerous cells in these tissue fragments, variably clustered throughout the sample (Additional file 1: Figure S1). The neuronal and glial nature of these cells was confirmed by immunohistochemical analyses using β3-Tubulin or NeuN, and GFAP monoclonal antibody, respectively (Fig. 2). In fact, β3-Tubulin (Fig. 2a) and NeuN (Fig. 2B and Additional file 1: Figure S1) staining from 10 μm-thick sections revealed the presence of many positive cells whereas GFAP staining (Fig. 2b and Additional file 1: Figure S1) highlighted an important astroglial network. In addition, V-GLUT1 (Fig. 2e) and GAD67 (Fig. 2f) immunostaining demonstrated the presence of glutamatergic and GABA-ergic neurons, respectively, and we also observed the presence of large TH-immunoreactive patches lacking nuclei.

These experiments confirm that DBS-obtained tissue fragments contain, besides astrocytes, neurons and fibers harbouring various neurotransmitter markers and reflect brain tissue of BG origin. We defined this collected material as brain tissue imprints (BTIs).

### Quantifying extracted proteins from STN and GPi-associated BTIs

**Fig. 3:**
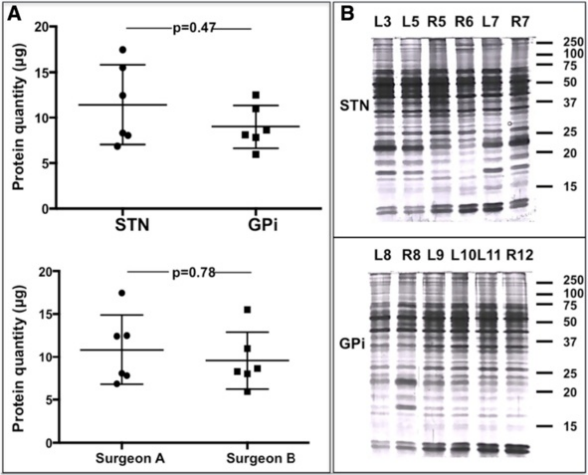
**a** BTI-extracted protein quantity. Scatter plot showing the quantity of proteins extracted from BTIs according to the targeted nucleus (up) and to the surgeon (bottom). **b** 1D SDS-PAGE electrophoresis of STN (top) and GPi (bottom) –associated BTIs. Two micrograms of proteins were loaded on 15 % polyacrylamide gel and revealed by silver staining

Before to envisage potential proteomic studies, it appeared important to determine the BTI-collected protein quantity, which, if too low, could be a limiting factor of our approach. For this purpose, 12 blunt stylets that were lowered in the STN (*n* = 6) or GPi (*n* = 6) by neurosurgeon A or B (Table 1) were collected. First, the Bradford protein assay of the 12 samples varied from 6.8 to 17.5 μg of proteins with a mean capture of 10.2 ± 3.6 μg of proteins. The comparison between STN and GPi-associated BTIs revealed no statistical difference between he different nuclei with 11.4 ± 4.4 and 8.9 ± 2.4 μg respectively (Mann Whitney test, *p* = 0.47) (Fig. 3a). In addition, we did not observe any significant difference in extracted-protein quantity according to the neurosurgeon who collected the BTIs. In fact, BTIs revealed 10.8 ± 4.1 and 9.6 ± 3.3 μg of proteins for neurosurgeon A and B, respectively (Mann Whitney test, *p* = 0.78), (Fig. 3a). These results suggest that BTIs can be consistently obtained in any electrode-implanted nucleus, are neurosurgeon-independent and contain amounts of proteins suitable for any proteomic protocols.

### Examining the detailed protein content of STN and GPi-associated BTIs by shotgun proteomics

To obtain a qualitative protein profile of these imprints, we separated by 1D electrophoresis both STN and GPi-associated BTIs (Fig. 3b). First, all samples revealed complex profiles with the presence of many protein bands on a wide mass range, from 14 to 250 kDa. Moreover, for both nuclei, the different BTIs revealed similar qualitative profiles with the presence of the same protein bands. Finally the comparison of STN and GPi-associated BTIs did not reveal obvious significant differences in protein profiles, showing a reproducible overall protein pattern. To further characterize STN and GPI-associated BTIs, 1D SDS-PAGE fractionation was combined with nano-LC-MS/MS analysis for extensive protein identification. Nano-LC-MS/MS analysis of STN-associated BTIs allowed the identification of 1’081 different proteins. The analysis of GPI-associated BTIs revealed 894 different proteins. Combining both protein lists highlighted a total of 1’298 distinct proteins (Additional file 2: Table S1). Among the 1’298 identified proteins, we observed neuron, astrocyte and oligodendrocyte-enriched proteins such as neuronal growth regulator 1, astrocytic phosphoprotein PEA-15 and myelin-oligodendrocyte glycoprotein (Table 2). We also identified proteins implicated in different nervous cell functions such as cell architecture, cell adhesion, synapse organization or neural maturation. Finally, consistent with immunofluorescence data detailed above, we also observed neurotransmitter-associated receptors and transporters such as glutamate receptor 2, metabotropic glutamate receptor 3 and vesicular glutamate transporter 1. These results confirmed the suitability of BTI to explore in depth the structural and functional proteome of different electrode-implanted nuclei.

**Table 2:** Brain-enriched and PD-associated proteins from BTIs. Table listing the accession number in Unirot database (ID), the isoelectric point (pI), the molecular weight (protein mass), and the name (description) of different brain-enriched or PD-associated proteins (bold)

Prot number	ID	Prot pI	Protein mass (Da)	Description
1	PEA15_HUMAN	4.9	15088	Astrocytic phosphoprotein PEA-15
2	BASP1_HUMAN	4.6	22562	Brain acid soluble protein 1
3	CNRP1_HUMAN	5.2	14224	CB1 cannabinoid receptor-interacting protein 1
4	GFAP_HUMAN	5.5	49880	Glial fibrillary acidic protein
5	GRIA2_HUMAN	7.6	96183	Glutamate receptor 2
6	PEBP1_HUMAN	7.8	21057	Hippocampal cholinergic neurostimulating peptide
7	LSAMP_HUMAN	5.7	31818	Limbic system-associated membrane protein
8	LYNX1_HUMAN	6.6	11937	Ly-6/neurotoxin-like protein 1
9	GRM3_HUMAN	7.7	96386	Metabotropic glutamate receptor 3
10	MBP_HUMAN	11.5	21493	Myelin basic protein
11	MYPR_HUMAN	8.8	29946	Myelin proteolipid protein
12	MOG_HUMAN	9.0	25110	Myelin-oligodendrocyte glycoprotein
13	NCAM1_HUMAN	4.8	92408	Neural cell adhesion molecule 1
14	NCAM2_HUMAN	5.5	91052	Neural cell adhesion molecule 2
15	L1CAM_HUMAN	5.8	137831	Neural cell adhesion molecule L1
16	NCALD_HUMAN	5.4	22114	Neurocalcin-delta
17	NCAN_HUMAN	5.2	140732	Neurocan core protein
18	NCDN_HUMAN	5.4	77243	Neurochondrin
19	NFASC_HUMAN	6.6	130112	Neurofascin
20	NFH_HUMAN	5.8	105639	Neurofilament heavy polypeptide
21	NFL_HUMAN	4.6	61385	Neurofilament light polypeptide
22	NFM_HUMAN	4.9	102341	Neurofilament medium polypeptide
23	NEUM_HUMAN	4.7	24803	Neuromodulin
24	NRCAM_HUMAN	5.7	130668	Neuronal cell adhesion molecule
25	NEGR1_HUMAN	6.5	31426	Neuronal growth regulator 1
26	GPM6A_HUMAN	5.7	29905	Neuronal membrane glycoprotein M6-a
27	GPM6B_HUMAN	5.9	36220	Neuronal membrane glycoprotein M6-b
28	SEPT3_HUMAN	6.5	40100	Neuronal-specific septin-3
29	NPTN_HUMAN	7.6	37792	Neuroplastin
30	NTRI_HUMAN	6.0	31738	Neurotrimin
31	SYN1_HUMAN	9.9	70033	Synapsin-1
32	SYN2_HUMAN	8.7	62847	Synapsin-2
33	SYN3_HUMAN	9.5	63303	Synapsin-3
34	SV2A_HUMAN	5.4	82695	Synaptic vesicle glycoprotein 2A
35	SNG3_HUMAN	8.8	24555	Synaptogyrin-3
36	SYNJ1_HUMAN	7.1	143254	Synaptojanin-1
37	SYPH_HUMAN	4.7	33845	Synaptophysin
38	SNP25_HUMAN	4.7	23315	Synaptosomal-associated protein 25
39	SYT1_HUMAN	8.4	47573	Synaptotagmin-1
40	VGLU1_HUMAN	7.6	61613	Vesicular glutamate transporter 1
41	VAMP1_HUMAN	8.7	12658	Vesicle-associated membrane protein 1
42	VAMP2_HUMAN	8.9	12532	Vesicle-associated membrane protein 2
43	GMFB_HUMAN	5.3	16582	Glia maturation factor beta
44	SYNPO_HUMAN	9.4	73666	Synaptopodin
45	SYUG_HUMAN	4.9	13330	Gamma-synuclein
46	SYUB_HUMAN	4.5	14288	Beta-synuclein
47	SYUA_HUMAN	8.8	11371	**Alpha-synuclein**
48	PARK7_HUMAN	6.8	19891	**Protein DJ-1**
49	UCHL1_HUMAN	5.5	24824	**Ubiquitin carboxyl terminal hydrolase isozyme L1**
50	VPS35_HUMAN	5.4	91707	**Vacuolar protein sorting-associated protein 35**

### Functional analysis of BTI-identified proteins

**Fig. 4:**
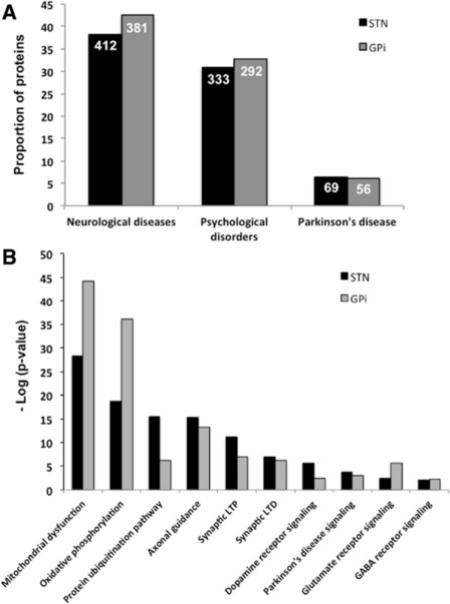
Functional analysis of the STN and GPi protein lists by Ingenuity Pathway Analysis. **a** Proportion of proteins associated to neurological, psychological and PD in STN and GPi protein lists, respectively. The data at the top of each bar correspond to the number of proteins associated to the disease. **b** Signalling pathways associated to brain functions and known to be altered in PD, and significantly represented in the STN and GPi protein lists (−log *p*-value > 1.3 i.e. *p*-value < 0.05)

To investigate the functional role of BTI-identified proteins, we separately submitted STN and GPi protein lists to Ingenuity Pathway Analysis Software (IPA) [23]. This automated annotation tool uses a knowledge database and assigns proteins to functional classes or specific canonical pathways related to various biological processes. To confirm the relevance of our BTI approach, we determined the relative representation of neurological diseases and brain-associated signalling pathways in STN and GPi-associated BTIs, respectively. First, the functional analysis revealed neurological diseases and psychological disorders as the two most represented disorders in both types of BTIs (Fig. 4a). In fact, STN and GPi protein lists respectively revealed 412 and 381 different species associated to neurological disease pathways, which represented 38.1 and 42.6 % of the total identified proteins in STN and GPi, respectively. Moreover, among these neurological disease-associated proteins, 69 and 56 species, which represented 6.4 and 6.3 % of the total identified proteins, were associated to PD in STN and GPi-BTIs, respectively. Finally, we also observed that canonical pathways (CPs), which were specific to brain functionality such as synaptic long term potentiation and neurotransmitter signalling pathways, or described as altered in PD such as mitochondrial dysfunction, oxidative phosphorylation and axonal guidance associated pathways were significantly represented (*p*-value < 0.05) in both types of BTIs (Fig. 4b). Of particular interest, PD signalling was significantly represented in both type of BTIs with the identification of several PD-relevant proteins such as α-synuclein, DJ-1 protein, UCHL1 protein and VPS35 protein, within which mutations have been associated with autosomal dominant or recessive forms of PD (Table 2). In addition, the proportion of proteins that was associated to these CPs were quite similar between the two types of BTIs. These results demonstrate the ability of the BTI approach to highlight brain and PD-related functional signatures in different nuclei and confirm that proteomics analysis of BTIs could be an appropriate strategy to study PD altered signalling pathways.

### Confirming the extraction of high-quality RNA from STN-associated BTIs

**Fig. 5:**
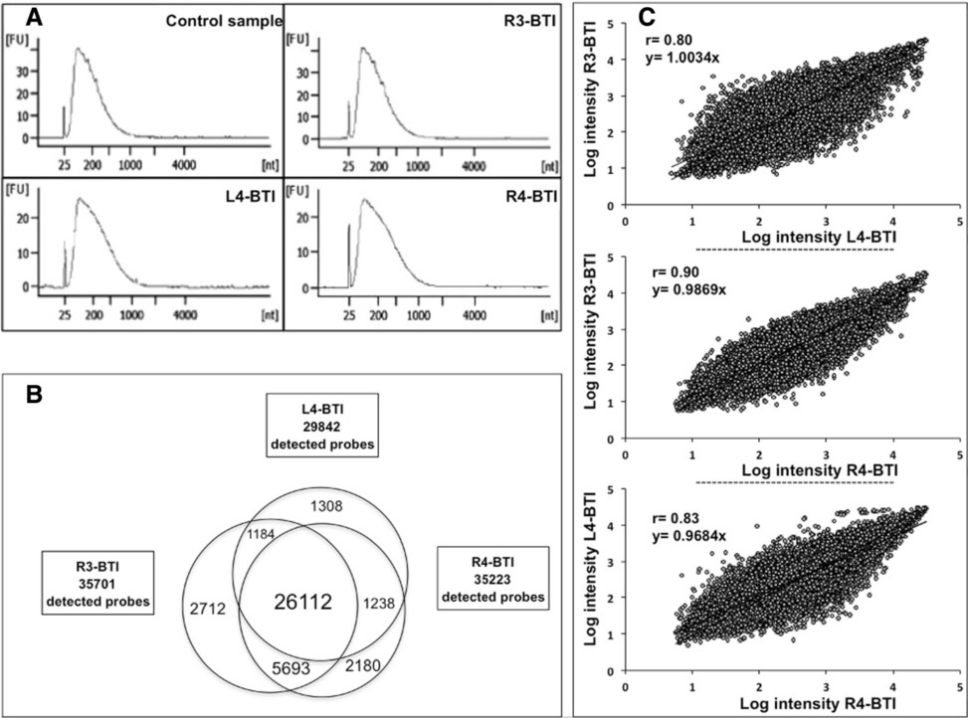
Transcriptomic analysis of BTI-extracted RNAs. **a** Quality control of BTI-extracted RNA by capillary electrophoresis migration. The electrophoretic profiles of the BTI-samples were compared with a high-quality control sample. **b** Microarray hybridization of the BTIs. Venn diagrams representing the number of detected and common probe sets in BTI samples by microarray analysis. **c** Intensity correlation for the common detected probe sets in the 3 BTIs. Linear regression curve representing the intensity of expression of common probe sets in the three BTIs. The coefficient of correlation (r) and the coefficient of the slope is dysplayed on the graphe

Given the major interest of gene expression profiling in PD-associated research, we assessed the capacity of BTIs to extract good quality RNAs from in vivo STN nucleus in PD patients. For this purpose, three different STN-stylets were collected, total RNA was extracted from these BTIs, and we applied a double amplification protocol to generate sufficient biological material for further analysis. After amplification rounds, these three BTIs revealed between 65 and 200 μg of RNAs, i.e. quantities appropriate for hybridization microarray analysis. To confirm the good quality of BTI-extracted RNAs, we applied the same preparation protocol on brain cortical sections obtained during epilepsy surgery and used as high-quality control sample. For this control sample, given the availability of sufficient starting material, the RNA quality was controlled and confirmed before amplification by capillary electrophoresis migration, which revealed full length RNAs and RNA integrity number (RIN) of 8.3 (Additional file 3: Figure S2). After a double amplification round, the control sample naturally revealed electrophoretic profile with shorter products that we compared with the BTI-obtained profiles. As shown on Fig. 5a, we observed a great similarity between BTI and control electrophoretic profiles with same length RNAs, implying that similarly to the control sample, BTI-collected RNAs were also full length and intact before amplification. These results demonstrate that high quality RNAs can be retrieved in vivo from DBS-targeted nuclei and are suitable for microarray analyses.

Given the sufficient quantity and the good quality of extracted RNAs, the samples were separately hybridized on microarrays to determine the complexity of expressed transcripts in BTI samples. Detailed results are available in Additional file 4: Table S2. The analysis of BTI-extracted RNAs highlighted from 29’842 to 35’701 different detected probe sets, which corresponded to a chip hybridization rate from 54.5 to 65 % (Fig. 5b). A chip hybridization rate of 60 % was obtained for the control sample, the analysis of which revealed 33’073 detected probe sets. Moreover the qualitative analysis of the BTI-associated chips revealed 26’112 common detected probe sets in the different BTIs, suggesting a robust qualitative reproducibility of the BTI approach. Moreover intensities of these common detected probe sets were strongly correlated between the different samples (Fig. 5c). In fact, the graphical representation of the 26’112 probe sets intensities in the different BTIs revealed a Pearson’s correlation coefficient of 0.8 to 0.9 with a slope value between 0.97 and 1.0, suggesting that most of these common probe sets were similarly expressed in the different BTIs, which originated from the same type of brain structure in the same disease condition. Altogether, these results demonstrate that high quality transcriptomic analyses may be undertaken from our BTI approach.

## Discussion

Accessing in vivo human brain samples for PD-associated research has long been considered an unrealistic approach, yet it may be one of the most relevant strategies to address such pivotal issues as etiopathogenic events, molecular pathways and diagnostic or therapeutic biomarkers. Since its first use in the ventral intermediate nucleus of the thalamus by Benabid et al. [24], DBS in PD has been extended to STN [25], GPi [26] and other deep brain nuclei [27], and has become the mainstay of surgical therapies to manage motor symptoms at early to advanced stages of the disease.

In the present study, we demonstrate for the first time that DBS-associated electrode implantation offers a unique opportunity to obtain in vivo fresh tissue imprints from relevant and electrophysiologically active deep brain structures. In fact, the stylet that is systematically used in DBS surgery, the tip of which is in immediate contact with the surrounding tissue, allows to recover, irrespective of brain targets and surgeons, tissue micro fragments that we have defined as BTIs. This unconventional material seems to be highly informative and of great scientific interest. Indeed, immunohistological analyses of STN-derived BTIs consistently showed populations of glutamatergic and GABAergic neurons, and the presence of TH-immunoreactive fibers, as expected in the STN-SN region [28]. Whereas the SN *pars reticulata* mainly contains large GABAergic projecting neurons, the STN is populated with glutamatergic neurons, some of which collateralizing locally and some receiving TH projections from SNpc-associated dopaminergic neurons [29]. Our findings are therefore in accordance with the current anatomical knowledge of these structures. Moreover, omic analyses of BTIs allowed to profile extensively their proteome and transcriptome, yielding lists of proteins and transcripts that turned out to be highly relevant for neurological diseases in general and PD in particular. Altogether, these preliminary results strongly support the concept of brain tissue imprinting during DBS surgery and the potential of this unique brain material to undergo informative poly-omic analyses.

The research paradigm proposed in this study is new and demonstrates many advantages over more conventional methods using post-mortem samples. First, the method of collecting BTIs is convenient and simple to perform in the context of DBS surgery, as it does not modify the DBS procedure. Second, this strategy proposes an in vivo access to deep brain nuclei and the collection of samples that are immediately processed in the surgical room within minutes after being captured, thus minimizing protein or RNA degradation [19, 20]. Moreover, BTIs are obtained in awaken and off-medication patients, according to the routine DBS procedure, thus limiting the potentially deleterious influence of anaesthesia and antiparkinsonian medication, respectively, on molecular metabolism. Third, BTIs being collected from highly selective brain structures, in this particular case BG nuclei, seem particularly relevant to address functional and structural issues related to the underlying pathology. As far as PD is concerned, electrode implantation usually occurs 10 to 12 years after PD diagnosis, and performing BTIs in this context would ensure the access to a homogeneous group of PD brains at an earlier stage of disease than post-mortem samples, which often derived from patients diagnosed 15 to 25 years prior to death. Furthermore, the recent use of DBS much earlier in the disease course, 5 to 7 years after diagnosis [30], opens the door to BTI-derived studies on molecular changes occurring only a few years after PD has started. Also, as DBS is currently exploring many other indications, including epilepsy, Alzheimer’s disease, obsessive-compulsive disorders, Gilles de la Tourette syndrome, depression, and many others, this approach may likely be transposed to any DBS-treated neurological [31] or psychiatric diseases [32]. Fourth, another advantage of our approach involves the number of samples that could be collected, which is virtually unlimited, as opposed to post-mortem samples which are notoriously difficult to obtain. Furthermore, if various conditions are operated, stratification of samples according to pathologies or nuclei, and statistically powerful comparisons of results across groups, one serving as control for the other may become possible.

The BTI method proposed here is in a preliminary phase and may have some limitations, yet solutions to circumvent them can be already considered. First, the biological material composing BTI samples is likely heterogeneous, involving various cell types, neuronal populations, dendrites, axons and other cellular extensions, synapses and other contacts between cells, blood vessels, extracellular fluid and so on, thus lacking cellular or subcellular specificity. While this may seem problematic, it may also be beneficial in providing a global view of the molecular state of the structure considered at a certain time point. Second, only existing surgical tools were used in this study, with the purpose of not modifying the routine procedure of DBS, yet it is possible that BTIs were contaminated by unwanted material during the descent of the stylet. In the future, new BTI-specific instruments and protocols may be designed to achieve an even more selective imprint and high-grade BTI purity. Third, whereas molecular analysis of STN and GPi-obtained BTIs may be of interest to investigate functional changes occurring in PD, other structures bearing alpha-synuclein pathology, like the SNpc, might be more relevant to address pathogenesis. Indeed, because the SN lies anatomically just below the STN, it is not unusual for the tip of the DBS electrode to be placed within the SN [33], and we therefore assume that BTIs can also be captured from this pivotal structure in certain patients.

Finally, at variance with post-mortem samples that can be collected both in diseased and normal states, DBS-associated BTIs can only be obtained in pathological, DBS-treatable conditions and will never concern healthy patients. To circumvent this important limitation, we propose to use a variety of non-degenerative conditions where DBS targeting of the same structures is also indicated. For example, PD-associated BTIs from STN, GPi or VIM may be compared with matching BTIs from non-PD conditions such as OCD and epilepsy (STN), dystonia and Gilles de la Tourette syndrome (GPi), and essential tremor (VIM) respectively [34–38]. Moreover, the application of DBS in PD patients with different disease durations, from 5 to 15 years may enable the stratification of BTIs according to disease duration and disease stages, and the comparison of early versus late biological changes potentially occurring in the basal ganglia. Finally, as PD is an asymmetrical disease and as BTIs could be collected bilaterally, comparisons may involve less versus more affected basal ganglia structures for each patient. Thus, several options are available to obtain comparative samples. While they may not totally fulfil the definition of control samples, they are likely to be sufficiently distinct from PD to allow robust and meaningful comparative analyses to be performed.

## Conclusion

In conclusion, this exploratory, proof-of-concept study proposes in vivo DBS-associated brain tissue imprinting as an original paradigm to explore the cellular and molecular profile of deep brain nuclei in human PD, and likely in any degenerative and non-degenerative DBS-treated neurological and psychiatric conditions. In particular, the biological material obtained through this approach appears to overcome most of the limitations inherent to post-mortem tissue and to be particularly suitable for omic research. Taking into account these aspects, BTI may become the ultimate sample of biological brain research in humans and significantly impact on future studies on pathogenesis, pathophysiology, biomarkers or therapeutic interventions in various neurological disorders.

## Methods

### Study participants

Twelve patients have been offered bilateral STN (*n* = 7) or GPi (*n* = 5) electrode implantation to alleviate their motor symptoms at the University Hospital of Grenoble. The STN-implanted group was 41 to 61 years (mean: 52.4 ± 7.1 years) and included five men and two women. The GPi-implanted group was 45 to 69 years (mean: 55.6 ± 8.9 years) and included three men and two women. Two different surgeons performed electrode implantations irrespective of the chosen target. Surgeon A performed 3 STN and 3 GPi electrode implantations. Surgeon B performed four STN and two GPi electrode implantations. Table 1 recapitulates the clinical and surgical characteristics of each patient.

Patients gave clear informed consent to participate in this study and we followed strict ethical guidelines given by the Comite Consultatif National d’Ethique (The French National Review Committee).

### Neurosurgical procedure

STN or GPi electrode implantations were performed with the ROSA stereotactic robot [22] (Medtech, Montpellier, France) at the University Hospital of Grenoble. Briefly, STN or GPi targeting was based on stereotactic 3 T magnetic resonance imaging (MRI) scan, which were imported into the planning station and reformatted into a plane parallel to the AC-PC plane by using robot planning software (ROSA from Medtech, Montpellier, France). This first step allowed determining an entry point while avoiding vessels of the cortical surface and the sulci. Then, under local anaesthesia, three to five parallel recording/stimulating microelectrodes each one into a guiding tube, and on a “BenGun” system were inserted into the brain using a motorized microdrive (Alpha-Omega, Nazareth, Israel) on a ROSA stereotactic robotic arm. The neuronal recording aimed to detect the target, based on its typical firing pattern and was followed by acute stimulation tests to determine the best site for the chronic electrode to be placed. Finally the chronic electrode (Medtronic, Minneapolis, USA) was implanted and connected to the pulse generator (Medtronic, Minneapolis, USA).

### Brain imprinting procedure

Once the best location for implantation of the definitive electrode has been defined, microelectrodes were removed from the guiding tube and replaced by a 1 mm-diameter, stainless steel blunt stylet (Dixi, Besancon, France), which slides through the guiding tube, protrudes by 1.5 mm out of it, and reaches the target in 10 to 15 s (Fig. 1a). The stylet tip was kept 1 min in contact with brain tissue to allow the imprinting process. To determine the location of the stylet tip into the brain during the imprint, intraoperative radiography (Fig. 1b) was performed and merged with the patient pre-operative MRI (Fig. 1c) using the robot planning software (ROSA from Medtech, Montpellier, France). The stylet was then retracted into the guiding tube so that the set can be withdrawn in 5 to 10 s without the stylet tip being contaminated during the removal step. Once the set had been removed from the brain, the stylet tip was immediately immersed in appropriate buffer for further analyses. From the 12 operated patients, a total of 19 different BTI samples were made available for the present study. BTIs could not be performed in 5 hemispheres due to technical constraints.

Nineteen different brain tissue imprints (BTI) had been collected by surgeon A (*n* = 10) and B (*n* = 9) and analysed by scanning electron microscopy (*n* = 1), immunohistochemistry and immunofluorescence (*n* = 3), proteomics (*n* = 12) and transcriptomics (*n* = 3) approaches. Table 1 recapitulates the type of analysis performed on each imprint.

### Scanning electron microscopy

One stylet was collected in the surgical room and tissue fragments that adhered to the stylet tip was collected in fixative solution, dehydrated through an ascending serie of ethanol solution and critical point dried using CO_2_. Tissue fragments were sputter-coated with gold prior to examination in a Cambridge S360 scanning electron microscope.

### Immunohistochemistry analyses

Stylets (*n* = 3) were collected in the surgical room and their tip was immediately immersed in fixative solution and cell block preparation was performed using the Shandon™ cytoblock™ preparation system (Thermo Fisher Scientific, MA, USA) according to the manufacturer’s protocol. Immuno-histofluorescence staining was performed on de-paraffinized BTIs 10 μm thick sections using antigen retrieval treatment and with secondary antibodies coupled with Alexa fluorochrome (A488 or A555). Neuronal and astroglial expression were identified using β3-tubulin (Covance, PRB435P) or NeuN (Millipore, MAB377) antibodies, and GFAP (DAKO, Z334) antibody, respectively. Further neuronal phenotypic characterization was investigated using antibody against: tyrosine hydroxylase (TH, Chemicon, AB152), vesicular glutamate transporter 1 for glutamatergic neurons (V-Glut1, Chemicon, MAB5502), glutamic acid decarboxylase-67 (GAD-67, Chemicon MAB5406) for GABA-ergic neurons. Sections were then counterstained with DAPI to label cell nuclei. Images were obtained using a fluorescence microscope Zeiss 5.1 (Zeiss, Iena, Germany) coupled to a digital camera and Axiovision 4.8 software (Zeiss).

### Proteomic analysis

#### Sample treatment

Stylets (*n* = 12) were collected in the surgical room and the stylet tip was immersed and kept under agitation 5 min at 4 °C in urea 8 M/CHAPS 2 % lysis buffer (Sigma-Aldrich, Saint-Louis, MO, USA). Samples were centrifuged 10 min at 10,000 g at 4 °C and the supernatant was recovered and frozen at −80 °C until analysis. For all samples, the delay between collection, protein extraction and freezing was less than 1 h to prevent protein degradation.

#### 1D SDS-PAGE electrophoresis

The total protein amount recovered from each BTI was quantified by the Bradford protein assay (Bio-Rad Laboratories, Hercules, CA, USA). Two micrograms of proteins from each BTI were individually mixed to Laemmli buffer from Bio-Rad. The mixture was heated in boiling water for 5 min and loaded on 15 % polyacrylamide gels. Staining was performed with 0.2 % silver nitrate solution for 2 min and protein bands were revealed in 0.02 % formaldehyde/ 0.005 % citric acid solution. Revelation was blocked with 1 % acetic acid solution and gels were scanned.

#### In-gel fractionation

For both STN and GPi, 2.5 μg of proteins from six different BTIs were pooled, and the resulting 15-μg total proteins were mixed to Laemmli buffer. The mixture was heated in boiling water for 5 min and loaded on a precast 4 to 20 % polyacrylamide gel from Bio-Rad. Staining and destaining were performed with Coomassie blue and 30 % ethanol/ 7 % acetic acid in water respectively. STN and GPI-associated lanes were cut into 12 homogeneous fractions that were washed in 100 mM ammonium bicarbonate for 15 min at 37 °C followed by a second wash in 100 mM ammonium bicarbonate, acetonitrile (1:1) for 15 min at 37 °C. Reduction and alkylation of cysteines were per- formed by mixing the gel pieces in 10 mM DTT for 35 min at 56 °C followed by 55 mM iodoacetamide for 30 min at room temperature in the dark. An additional cycle of washes in ammonium bicarbonate and ammonium bicarbonate/acetonitrile was then performed. Proteins were digested by incubating each gel slice with modified sequencing grade trypsin (Promega) in 50 mM ammonium bicarbonate overnight at 37 °C. The resulting peptides were extracted from the gel by three steps: a first incubation in 50 mM ammonium bicarbonate for 15 min at 37 °C and two incubations in 10 % formic acid, acetonitrile (1:1) for 15 min at 37 °C. The three collected extractions were pooled with the initial digestion supernatant, dried in a SpeedVac, and resuspended with 12 μl of 5 % acetonitrile, 0.1 % formic acid.

#### Nano-LC-MS/MS analysis

Protein identification of each fraction was performed by nano-liquid chromatography tandem mass spectrometry (nano-LC-MS/MS) and each fraction was analysed twice to obtain technical replicates and to increase protein identification.

Nano-LC-MS/MS was performed on a linear trap quadrupole (LTQ) Orbitrap Velos Pro (Thermo Electron, San Jose, CA, USA) equipped with a NanoAcquity system (Waters). Peptides were trapped on a home-made 5 μm 200 Å Magic C18 AQ (Michrom) 0.1 × 20 mm pre-column and separated on a commercial 0.075 × 150 mm Nikkyo (Nikkyo Technology) analytical nanocolumn (C18, 5 μm, 100 Å). The analytical separation was run for 65 min using a gradient of H_2_O/FA 99.9 %/0.1 % (solvent A) and CH_3_CN/FA 99.9 %/0.1 % (solvent B). The gradient was run as follows: 0–1 min 95 % A and 5 % B, then to 65 % A and 35 % B for 55 min, and 20 % A and 80 % B for 65 min at a flow rate of 220 nL/min. For MS survey scans, the orbitrap (OT) resolution was set to 60000 and the ion population was set to 5 × 10^5^ with an m/z window from 400 to 2000. For protein identification, up to eight precursor ions were selected for collision-induced dissociation (CID) in the LTQ. The ion population was set to 1 × 10^4^ (isolation width of 2 m/z) while for MS/MS detection in the OT, it was set to 1 × 10^5^ with an isolation width of 2 m/z units. The normalized collision energies were set to 35 % for CID.

#### Peptide and protein identification

MS data were processed using EasyProtConv. For each imprinted nucleus, the peak list of each fraction was generated from raw data CID spectra using the EasyProtConv conversion module. The peak lists of all fractions were then merged into a single peak list file per nucleus on EasyProtConv. The two different obtained peak lists were submitted to Easyprot, a platform that uses Phenyx (GeneBio, Geneva, Switzerland) for protein identification [39]. Searches were conducted against UniProt Swiss-Prot database (669 903 entries on October, 2014) specifying Homo sapiens taxonomy. Specificity of trypsin digestion was set for cleavage after Lys or Arg except before Pro, and two missed trypsin cleavages were allowed. Carbamidomethylation of cysteines was set as a fixed modification, and oxidation of methionine was set as variable modifications. Peptide z-scores were then set to maintain a false positive peptide ratio below 1 %. Proteins with at least two distinct peptide sequences were automatically validated. Proteins were clustered based on shared peptides, with the protein entry containing the most peptides selected as the group reporter.

#### Functional analysis by ingenuity pathway analysis

The STN and GPI-associated protein lists were further submitted to Ingenuity Pathway Analysis software (Ingenuity Systems, Redwood City, CA) to perform the functional characterization of the proteins recovered from the BTIs. This automatic annotation tool uses a knowledge database and assigns proteins to functional classes or specific canonical pathways (CPs) related to various biological processes [23].

### Transcriptomic analysis

#### RNA extraction, amplification and quality control

STN-associated BTIs (*n* = 3) were collected in the surgical room and quickly immersed and kept under agitation 5 min in 100 μl lysis buffer from the mirVana isolation kit™ (Ambion, Applied Biosystems, Foster City, CA) to prevent RNase activity and therefore to limit RNA degradation. In parallel, two 60 μm-thick sections from a cortical piece, which was obtained during a previous surgical tissue resection in an epilepsy patient, were cut at −20 °C and immediately immersed in 100 μl lysis buffer. This sample was used as a technical control. After tissue lysis, total RNA was isolated from BTI and technical control using a phenol:chloroform extraction protocol (MirVana isolation kit™, Ambion, Applied Biosystems). Before RNA amplification, the quality and integrity of the technical control were confirmed (RNA integrity = 8.3, suppl data 2) by capillary electrophoresis migration using RNA nano 6000 kit and the Bioanalyzer 2100 (Agilent Technologies, Palo Alto, CA). Then total RNAs from BTIs and technical control were separately amplified with the GeneChip 3’ IVT Express Kit. First, total RNAs were reverse-transcribed using primers polydT-T7 promoter sequence; cDNAs were double-stranded synthesized and finally amplified by the T7 RNA polymerase. A second amplification was performed with WT-expression kit™ (Ambion, Applied Biosystems, Foster City, CA) to obtain biotinylated cDNA. After this double amplication procedure, the RNA concentration of each BTI sample was determined with the measure of absorbance at 260 nm, and the electrophoretic profiles of BTI-samples were compared to the technical control to indirectly confirm the quality of BTI-extracted RNA.

#### Microarray hybridization

Microarrays experiments were carried out in accordance with the protocol GeneChip Expression Wash, Stain and Scan from Affymetrix (Santa Clara, CA, USA). Briefly, 15 μg of labeled cRNA was hybridized for 16 h at 45 °C on GeneChip Human Genome U133 Plus 2.0 (Affymetrix, Santa Clara, CA, USA) corresponding to 54675 unique gene probe sets. The arrays were washed and stained with streptavidin − phycoerythrin before scanning. The fluorescence values of each probe set signal, reported in arbitrary units, were processed with MAS5 statistical algorithm to validate the probe set signal “absent” or “present”, and finally they were normalized between all the arrays using the Robust Multichip Analysis (RMA) algorithm. These algorithms are included in Affymetrix expression console (Affymetrix, Santa Clara, CA, USA).

## Additional files

Additional file 1: Figure S1. BTI-associated immunohistochemical analyses. Immunostaining of the dilator-bound material (X200) revealed (top) the presence of many cells by hematoxylin-eosin staining among which (middle) neurons by neuronal nuclei (NeuN) staining and (bottom) astrocytes by glial fibrillary acid protein (GFAP) staining. (DOCX 551 kb) Additional file 2: Table S1. Non-redundant lists of the proteins identified in STN or GPi-associated BTIs. (XLSX 401 kb) Additional file 3: Figure S2. Confirmation of the technical control-related RNA Integrity by capillary electrophoresis. (DOCX 191 kb) Additional file 4: Table S2. Micro-array hybridization results. The table recapitulates detected (P) and non-detected (A) transcripts in all analyzed samples. (XLSX 12765 kb)
