# Supplementary material for: Deep brain stimulation-associated brain tissue imprints: a new in vivo approach to biological research in human Parkinson’s disease
Source: Mol Neurodegener. 2016 Jan 28;11:12. doi: 10.1186/s13024-016-0077-4 (PMC4730746; doi:10.1186/s13024-016-0077-4)
Supplement: Additional file 1: Figure S1. — BTI-associated immunohistochemical analyses. Immunostaining of the dilator-bound material (X200) revealed (top) the presence of many cells by hematoxylin-eosin staining among which (middle) neurons by neuronal nuclei (NeuN) staining and (bottom) astrocytes by glial fibrillary acid protein (GFAP) staining. (DOCX 551 kb) [file 13024_2016_77_MOESM1_ESM.docx]

**BTI-associated immunohistochemistry analysis**.

4 μm thick sections were analyzed by immunohistochemistry using mouse monoclonal anti-human GFAP (Dako, Z0334, 2.9 g/l) and mouse monoclonal anti-NeuN (Chemicon, MAB377, 1mg/ml). All stainings were performed on the Ventana Discovery XT automated system (Ventana Medical Systems, Tucson, AZ, USA) and Ventana reagents for the entire procedure were used. For GFAP antibodies, no antigen retrieval pre-treatment was required, whereas for NeuN antibodies slides were heated 36 min in CC1 cell conditioning solution to retrieve antigenicity. Slides were incubated 30 min at 37^o^C with primary antibodies respectively diluted at 1/2000 (GFAP) and 1/200 (NeuN) in antibody diluent from Ventana. Detection of primary antibodies was carried out using the secondary universal biotinylated antibodies reagent and the DABMap detection kit (Ventana Medical Systems), based on conversion of diaminobenzidine to a dye with multimeric horseradish peroxidase (HRP).


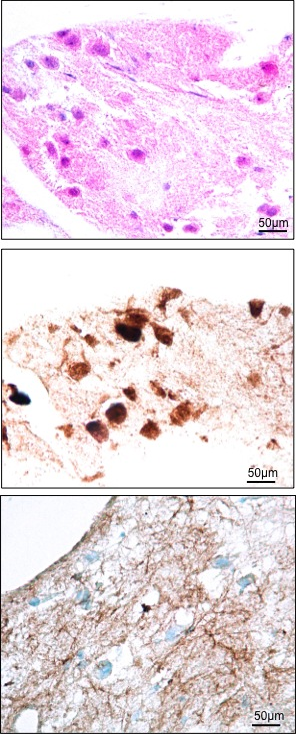


Figure 1: **BTI-associated immunohistochemical analyses.** Immunostaining of the dilator-bound material (X200) revealed (top) the presence of many cells by hematoxylin-eosin staining among which (middle) neurons by neuronal nuclei (NeuN) staining and (bottom) astrocytes by glial fibrillary acid protein (GFAP) staining.
