# Supplementary material for: Deep brain stimulation-associated brain tissue imprints: a new in vivo approach to biological research in human Parkinson’s disease
Source: Mol Neurodegener. 2016 Jan 28;11:12. doi: 10.1186/s13024-016-0077-4 (PMC4730746; doi:10.1186/s13024-016-0077-4)

Quality and Integrity of RNA in the control sample obtained from *in vivo* cortical resection. Both graphics demonstrate the full lenght of RNA (nt: nucleotide number) in this sample before double amplification. And the RNA Integrity number revealed a value of 8.3 and thus confirmed the good quality of this sample.

**RNA Integrity Number: 8.3**


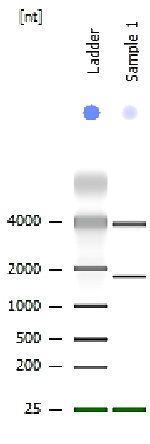


**4000**

**2000**

**1000**

**500**

**200**

**25**

**Ladder**

**Control sample**

**nt**


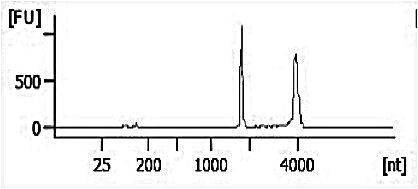

Supplement: Additional file 3: Figure S2. — Confirmation of the technical control-related RNA Integrity by capillary electrophoresis. (DOCX 191 kb) [file 13024_2016_77_MOESM3_ESM.docx]
